# Human induced neural stem cells support functional recovery in spinal cord injury models

**DOI:** 10.1038/s12276-023-01003-2

**Published:** 2023-06-01

**Authors:** Daryeon Son, Jie Zheng, In Yong Kim, Phil Jun Kang, Kyoungmin Park, Lia Priscilla, Wonjun Hong, Byung Sun Yoon, Gyuman Park, Jeong-Eun Yoo, Gwonhwa Song, Jang-Bo Lee, Seungkwon You

**Affiliations:** 1 Laboratory of Cell Function Regulation, Department of Biotechnology, College of Life Sciences and Biotechnology, Korea University, Seoul, 02841 Republic of Korea; 2 Institute of Animal Molecular Biotechnology, College of Life Sciences and Biotechnology, Korea University, Seoul, 02841 Republic of Korea; 3 Institute of Regenerative Medicine, STEMLAB, Inc., Seoul, 02841 Republic of Korea; 4 Institute of Future Medicine, STEMLAB, Inc., Seoul, 02841 Republic of Korea; 5 Department of Neurosurgery, College of Medicine, Korea University Anam Hospital, Seoul, 02841 Republic of Korea

**Keywords:** Neural stem cells, Spinal cord diseases

## Abstract

Spinal cord injury (SCI) is a clinical condition that leads to permanent and/or progressive disabilities of sensory, motor, and autonomic functions. Unfortunately, no medical standard of care for SCI exists to reverse the damage. Here, we assessed the effects of induced neural stem cells (iNSCs) directly converted from human urine cells (UCs) in SCI rat models. We successfully generated iNSCs from human UCs, commercial fibroblasts, and patient-derived fibroblasts. These iNSCs expressed various neural stem cell markers and differentiated into diverse neuronal and glial cell types. When transplanted into injured spinal cords, UC-derived iNSCs survived, engrafted, and expressed neuronal and glial markers. Large numbers of axons extended from grafts over long distances, leading to connections between host and graft neurons at 8 weeks post-transplantation with significant improvement of locomotor function. This study suggests that iNSCs have biomedical applications for disease modeling and constitute an alternative transplantation strategy as a personalized cell source for neural regeneration in several spinal cord diseases.

## Regenerative medicine: stem cell opportunity for spinal cord repair

Neural stem cells (NSCs) directly converted from readily accessible cell populations can successfully initiate tissue repair in rodent models of severe spinal cord injury. While NSCs as a regenerative therapy are of increasing interest, they are difficult to obtain. Daryeon Son of Korea University, Seoul, South Korea, and colleagues have now demonstrated that highly accessible human urine-derived cells can be reprogrammed into induced NSCs (iNSCs). The researchers induced two types of severe spinal cord injury in rats and then transplanted human iNSCs to the damaged sites. Within months, they observed considerable tissue repair, including extensive axonal growth, the formation of synaptic connections between host and graft neurons, and improvement in locomotor function. iNSCs could create new opportunities for treating a range of currently untreatable traumatic injuries.

## Introduction

Neural stem cell (NSC) transplantation is an attractive therapeutic approach for the management of spinal cord injury (SCI)^1–3^. NSCs are self-renewing, multipotent cells that can be maintained and expanded in vitro for a long time and differentiated into committed neural subtypes, such as neurons, astrocytes, and oligodendrocytes^4^. The past decade of research has shown that transplantation of NSCs into the lesion sites of SCI contributes to regeneration of neural lineage subpopulation cells, extension of large numbers of axons, and formation of functional synaptic connectivity with host circuits over long distances^5–10^. In addition, grafted NSCs can differentiate into functional oligodendrocytes, promoting remyelination of axons around the injury sites^11–13^.

Although NSCs can be isolated primarily from fetal and adult human tissues, the scarcity of donors, ethical concerns, and immunogenicity limit their applications^14–17^. Neural differentiation from pluripotent stem cells (PSCs), including embryonic stem cells (ESCs) and induced pluripotent stem cells (iPSCs), is a more feasible approach to obtain NSCs for the treatment of SCI^18^. Nonetheless, there are concerns regarding the clinical use of ESCs and iPSCs, such as ethical controversy, immunogenicity, and tumorigenicity. Direct reprogramming of somatic cells, known as transdifferentiation, provides benefits over ESCs and iPSCs in terms of tumorigenicity risk, patient specificity, and time and labor efficiency. Accumulated knowledge of cell fate specification and plasticity has allowed the direct reprogramming of somatic cells into lineage-restricted stem cells while bypassing an intermediate pluripotency state^19,20^. The resulting functional cells are expected to be more widely utilized as a complementary tool to PSC techniques in personalized therapy and stem cell transplantation for SCI.

Despite encouraging results from preclinical and early-stage clinical trials focusing on stem cell transplantation for SCI, stem cell therapies have shown highly variable efficacies, which may be mainly attributable to poor survival and engraftment in host tissues after transplantation^5,8–10,21–23^. Transplanted cells are exposed to a harsh environment with secondary pathologies, including oxidative stress, hypoxia, inflammation, and neuronal injury. The vast majority die via necrosis and apoptosis at the transplantation site^24,25^. One strategy to protect transplanted cells against external stresses and enable them to replace a damaged or missing part of the remaining tissue is to use a hydrogel-mediated delivery system. In this system, cells are immobilized in a three-dimensional polymer network surrounded by a semipermeable membrane, allowing the localized and sustained release of trophic factors to drive differentiation and regeneration^5,6,26,27^.

Previously, we proposed a simple and time-efficient method to directly generate induced NSCs (iNSCs) from human urine cells (UCs), which can be isolated safely, inexpensively, and noninvasively, by employing a self-replicative mRNA system with controllable expression of OCT4, KLF4, SOX2, and GLIS1 (OKSG)^28^. For the generation and clinical application of iNSCs to treat SCI, we sought to generate iNSCs from patient somatic cells. In the present study, we generated iNSCs with high reproducibility and efficiency and reported their engraftment into two SCI rat models. Graft-derived neurons extended large numbers of axons over long distances and subsequently reestablished host-to-graft and graft-to-host connectivity at 8 weeks posttransplantation, contributing to restoration of synaptic connections across the injured spinal cord and a significant improvement in locomotor function. These iNSCs may provide an alternative approach to generate the optimal cell types for therapeutic transplantation in patients with spinal cord diseases.

## Materials and methods

### Generation of iNSCs from human somatic cells and hESC-derived NSCs

This study was approved by the Institutional Review Board at Korea University (IRB approval number; KUIRB-2020-0278-01). iNSCs were directly converted from human UCs, BJ fibroblasts (ATCC, Rockville, MD, USA) and patient fibroblasts (GM01503, GM03672, and GM13411, Coriell Institute for Medical Research, Camden, NJ, USA) by synthetic mRNAs, including OCT4, KLF4, SOX2, GLIS1, and B18R, followed by culture in chemically defined medium as previously described^28^. Briefly, 1 × 10^6^ somatic cells were transfected with synthetic mRNAs by electroporation (MP-100, Thermo Fisher Scientific, Waltham, MA, USA). At day two after transfection, UCs were reseeded on Matrigel (BD Biosciences Clontech, Palo Alto, CA, USA)-coated plates with B18R protein and cultured in medium consisting of DMEM/F12: Neurobasal (Thermo Fisher Scientific) (1:1), 1 × N2 (Thermo Fisher Scientific), 1 × B27 (Thermo Fisher Scientific), 1x penicillin/streptomycin, 1 × l-glutamine, 1 × nonessential amino acids, 10 ng/mL recombinant human LIF (MilliporeSigma, Burlington, MA, USA), 2 μM SB431542 (Tocris Bioscience, Missouri, UK) and 3 μM CHIR99021 (Tocris Bioscience) (LSC medium) with small molecules (0.5 μM purmorphamine (Tocris Bioscience), 10 μM forskolin (Tocris Bioscience), 64 μg/mL vitamin C (MilliporeSigma), and 100 μM sodium butyrate (Tocris Bioscience)). At 12 days after induction, neuroepithelial-like iNSC colonies were picked up and transferred onto Matrigel-coated plates in LSC medium. iNSCs were expanded in LSC medium and subcultured using Accutase (STEM CELL Technologies, Vancouver, BC, Canada). To generate GFP-expressing iNSCs, cells were infected with a lentiviral vector encoding GFP^28^. For induction of NSCs from hESCs (WA14 ESCs), hESCs were treated with LSC medium on Matrigel-coated plates for 2 weeks^29^.

### In vitro differentiation

For spontaneous differentiation into neurons, iNSCs were seeded on poly-L-ornithine (PLO)/Laminin-coated plates and cultured in a differentiation medium consisting of DMEM/F12, 1 × N2, 1 × B27, 300 ng/mL cAMP (Tocris Bioscience), 64 μg/mL Vitamin C, 10 ng/mL BDNF (Peprotech, Rocky Hill, NJ, USA), and 10 ng/mL GDNF (Peprotech) for 2 weeks. For GABA neuron differentiation, iNSCs were seeded on PLO/Laminin-coated plates and cultured in differentiation medium consisting of DMEM/F12, 1 × N2, 0.5 × B27, and 300 ng/mL cAMP (Tocris Bioscience) for 3 weeks. For motor neuron differentiation, iNSCs were seeded on PLO/laminin-coated plates and cultured in a posterior hindbrain- and spinal cord-specified medium consisting of DMEM/F12, 1 × N2, 1 × B27, 20 μg/mL insulin (MilliporeSigma), 10 ng/mL FGF2, 10 ng/mL EGF, 1 μM retinoic acid, and 1 μg/mL SHH for 1 week and, after removal of FGF2 and EGF, cultured for another 1 week. Finally, cells were differentiated in DMEM/F12, 1 × N2, 1 × B27, 20 ng/mL BDNF, 20 ng/mL GDNF, and 50 ng/mL SHH for 2 weeks. For astrocyte differentiation, iNSCs were seeded on Matrigel-coated plates and cultured in differentiation medium consisting of DMEM/F12, 1 × N2, 1 × B27, 20 ng/mL CNTF (Peprotech), and 10 ng/mL BMP4 (Peprotech) for 1–2 weeks.

### RT‒PCR and Real-time PCR

Total RNA was isolated from cells with TRIzol (Invitrogen, Carlsbad, CA, USA), and cDNA was synthesized using AccuPower^®^ RT-PreMix (Bioneer, Daejeon, South Korea) with oligo-dT-18 primers (Bioneer). Target gene fragments were amplified by specific primers (Supplementary Table 1). GAPDH was used as a housekeeping gene (normalization control). qRT-PCR was performed with iQ SYBR Green Supermix (Bio-Rad, Hercules, CA, USA). The primer sequences used in this study are listed in Supplementary Table 1.

### Immunocytochemistry

Cells were fixed in 4% paraformaldehyde (PFA) for 20 min at room temperature and placed in PBS containing 0.3% Triton X-100 and 5% donkey serum for 20 min at room temperature. Next, the cells were exposed to primary antibodies (Supplementary Table 2) at 4 °C overnight and then secondary antibodies for 1 h at room temperature. Nuclei were stained with DAPI for 5 min at room temperature. Immunofluorescence images were acquired by a fluorescence microscope (IX71, OLYMPUS, Tokyo, Japan).

### Karyotype analysis

Karyotyping was conducted by GTG banding by Samkwang Medical Laboratories (Seoul, South Korea).

### Cell line authentication

Somatic cells and iNSCs were authenticated using STR analysis by Cosmo Genetech (Seoul, South Korea).

### Mycoplasma contamination detection

The absence of mycoplasma contamination of iNSCs was confirmed by a MycoAlert^TM^ PLUS Mycoplasma Detection kit (Lonza, Basel, Switzerland).

### Characterization of fibrin hydrogels and cytotoxicity

Fibrinogen (MilliporeSigma) and thrombin (MilliporeSigma) solutions were prepared using Tris-buffered saline (pH 7.4), sterile filtered, and diluted to appropriate concentrations. Injectable fibrin hydrogels were prepared by combining fibrinogen and thrombin solutions. The time required for gel formation was investigated using the standard vial inversion technique following the addition of thrombin (1, 3, and 5 U/ml final concentration) to fibrinogen solutions (5, 10, 20, and 30 mg/ml final concentration)^30^. The cross-sectional morphologies of fibrin hydrogels were determined using a scanning electron microscope (Quanta 250 FEG; FEI, OR, USA). The viability of iNSCs entrapped in fibrin hydrogels was determined by fluorescent staining with calcein/EthD (Live/Dead Cytotoxicity Kit; Invitrogen) and LDH leakage (BIOMAX, Seoul, South Korea) after incubation for 24 h according to the manufacturer’s instructions. LDH release (cytotoxicity) was calculated using the following equation:

$$Cytotoxicity\,\left( \% \right) = \frac{{test\,sample - low\,control}}{{high\,control - low\,control}} \times 100$$

### SCI

The animal study was approved by the Institutional Animal Care and Use Committee of the Korea University College of Medicine (approval number: KOREA-2020-0021). Seven-week-old female Sprague–Dawley (SD) rats and nude rats (Crl:NIH-*Foxn1*^*rnu*^) were used. They were anesthetized with 5% isoflurane and placed in an induction chamber filled with 3% isoflurane before surgery. After laminectomy of T3, animals underwent crushing with forceps for 10 s (SD rats) or complete transection (nude rats). All animals received subcutaneous injection of antibiotics (cefazolin, 10 mg/kg) and analgesia (ketorolac tromethamine, 1 mg/kg) for 2 days after surgery. Bladders were manually emptied twice per day until urination function was restored.

### Cell transplantation

Two weeks after SCI, 1.5 × 10^6^ cells were resuspended in 10 μl of fibrin gel containing a growth factor cocktail, prepared by mixing 25 mg/ml fibrinogen (MilliporeSigma) and 25 U/ml thrombin (MilliporeSigma) containing growth factors BDNF (50 μg/ml, Peprotech), VEGF (10 μg/ml, Peprotech), bFGF (10 μg/ml, Peprotech) and MDL28170 (50 μM, MilliporeSigma)^26^ and immediately transplanted into the lesion epicenter (depth: 1 mm) using a Neuros Syringe (Hamilton, Reno, NV, USA) and Quintessential Stereotaxic Injector (Stoelting, Wood Dale, IL, USA). After cell transplantation, immunosuppressants (cyclosporin A, 15 mg/kg) were administered daily to SD rats by subcutaneous injection. Animals survived and were sacrificed at 8 weeks post cell transplantation. For axonal tract tracing, 5 μl of biotinylated dextran amine (10%, BDA; 10,000 MW, Invitrogen, Carlsbad, CA, USA) was injected in the rostral area at the lesion site 2 weeks before perfusion.

### Histology and immunohistochemistry

Animals were perfused with 4% PFA at 8 weeks after cell transplantation. The spinal cords were dissected out and postfixed at 4 °C overnight, placed in 30% sucrose for 3 days and embedded in OCT compound (Sakura, Torrance, CA, USA). Tissues were sectioned on a cryostat (CM1950, Leica, Wetzlar, Germany) at a thickness of 30 μm. For antigen recovery, the sectioned tissues were incubated in PBS containing 1% SDS (sodium dodecyl sulfate) for 5 min. Sections were incubated with primary antibodies (Supplementary Table 2) at 4 °C overnight and then stained with Alexa Fluor 488- and 594-conjugated donkey secondary antibodies (Invitrogen) for 2.5 h at room temperature. For BDA tract tracing, tissue samples were incubated with Alexa Fluor 488-conjugated streptavidin (Invitrogen). Nuclei were stained with DAPI for 5 min at room temperature. Sections were covered with mounting medium (Fluoromount-G, Invitrogen, 00-4958-02) and glass coverslips. Immunofluorescence was visualized using a fluorescence microscope (IX71) and confocal laser scanning microscope (LSM 700, Zeiss, Jena, Germany).

### Behavior analysis

Hindlimb locomotion was assessed using the Basso, Beattie, and Bresnahan (BBB) locomotor rating scale in an open field and scored once per week over 10 weeks after injury.

### Statistical analysis

Experimental data are expressed as the mean ± standard deviation (SD) of >three replicates. Data comparisons were performed by one-way ANOVA with Tukey’s *post hoc* test for multiple comparisons. Differences with *p < 0.05, **p < 0.01, *and* ***p < 0.001 were considered statistically significant.

## Results

### Direct reprogramming of human somatic cells into iNSCs

**Fig. 1:**
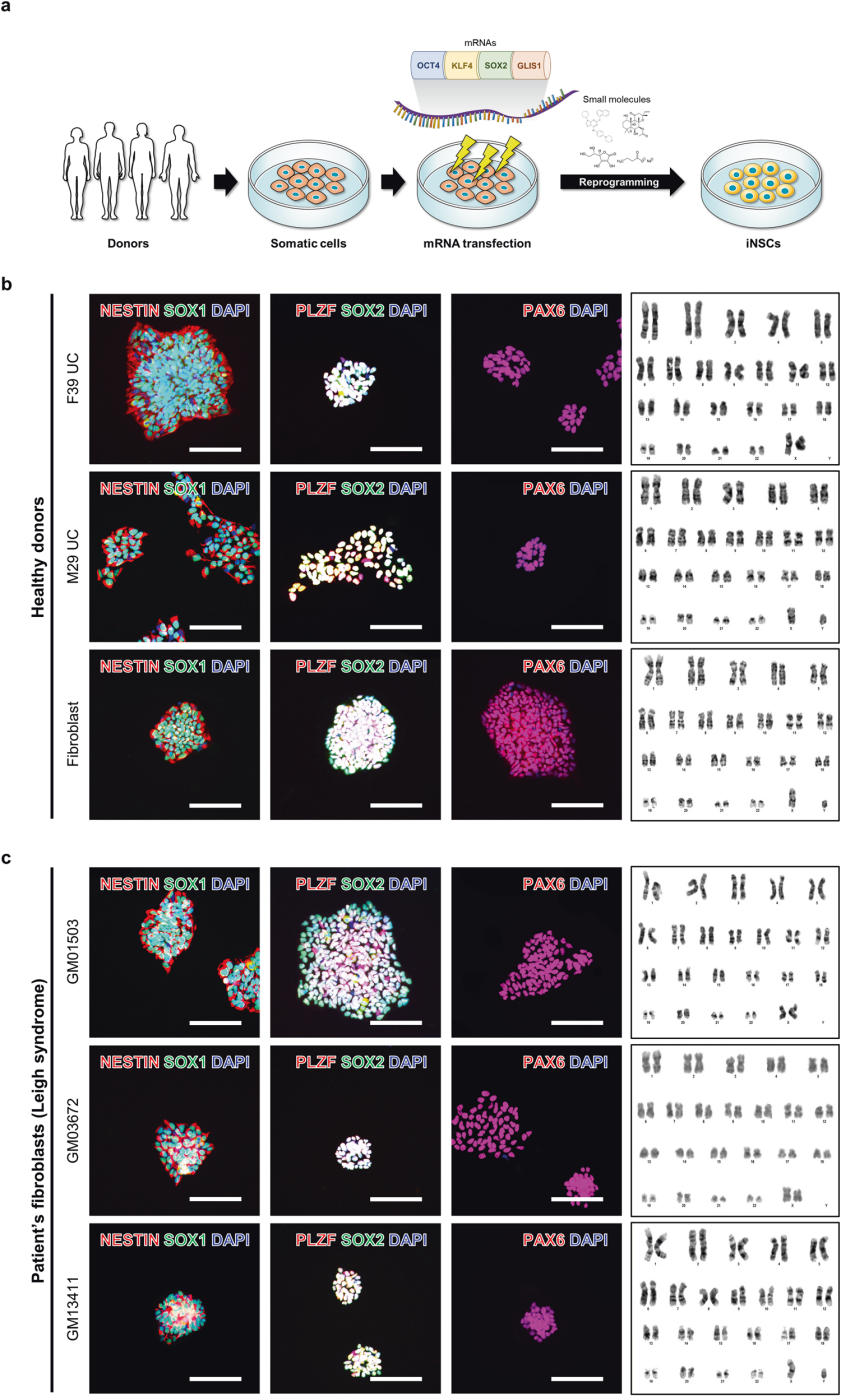
Direct reprogramming of human somatic cells into induced neural stem cells (iNSCs). **a** Schematic diagram of the experimental procedure. **b**, **c** Expression of neural stem cell (NSC) markers (NESTIN, SOX1, PLZF, SOX2, and PAX6) and karyotyping of iNSCs. Scale bars, 200 μm (**b**, **c**).

Our previous work demonstrated that iNSCs can be generated from UCs in a safe and time- and labor-effective manner using the self-replicative mRNA OKSG. These iNSCs have a posterior-dorsal phenotype and can differentiate into astrocytes, oligodendrocytes, and different types of neurons^28^. In the present study, to explore whether this method is applicable to human somatic cells, we attempted to establish iNSC lines from human UCs (female and male), commercial fibroblasts (BJ cells), and fibroblasts obtained from patients (Fig. 1a). It has been reported that there are two types of urinary cell colonies (Type 1 and 2)^31–33^. In our results, the major and surviving cell types of UCs from healthy donors are type 2 cells (Supplementary Fig. 1)^31^. All donated cells and BJ fibroblasts could be directly converted into iNSCs with high reproducibility and efficiency. The resulting iNSC lines expressed NSC-specific markers such as NESTIN, SOX1, PLZF, SOX2, and PAX6 and had a normal karyotype (Fig. 1b, c, Supplementary Fig. 2). These results demonstrate that this method can be used to generate iNSCs from various somatic cells in a safe and time- and labor-effective manner.

### In vitro characterization of GFP-expressing iNSCs

**Fig. 2:**
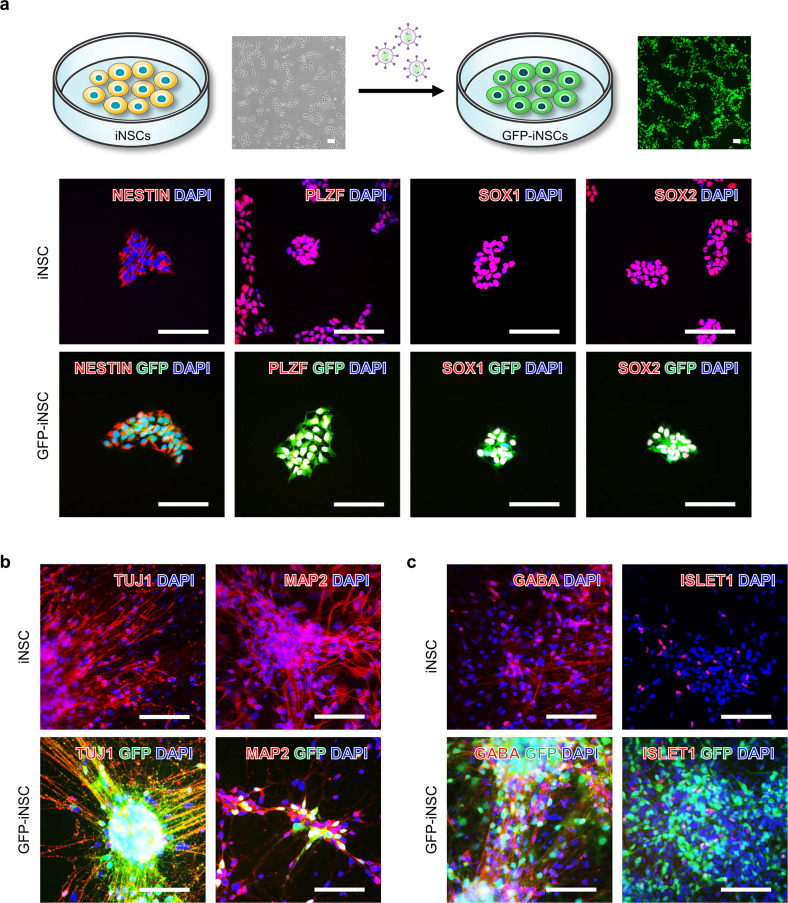
Characterization of iNSCs and GFP-iNSCs in vitro. **a** Expression of NSC markers (NESTIN, PLZF, SOX1 and SOX2) in iNSCs and GFP-iNSCs. **b** Expression of TUJ1 and microtubule-associated protein 2 (MAP2) in differentiated iNSCs and GFP-iNSCs. **c** Expression of various subtypes of mature neuron markers, including gamma-aminobutyric acid (GABA)ergic and ISLET1 (motor) neurons, in differentiated iNSCs and GFP-iNSCs. Scale bars, 200 μm (**a**–**c**).

Morphological changes, behaviors, cell‒cell linkages, migration, and localization of GFP-expressing NSCs were monitored in vitro/in vivo^6,9,26,34,35^. To evaluate the regenerative capacity of iNSCs after SCI, the generated iNSCs were transduced with a GFP-expressing lentiviral vector (referred to as GFP-iNSCs) (Supplementary Fig. 3a). The expression of NSC markers (NESTIN, PLZF, SOX1, and SOX2) did not markedly differ between iNSCs and GFP-iNSCs, and their differentiation potential into TUJ1^+^ and MAP2^+^ (immature and mature markers) neurons was similar (Fig. 2a, b, Supplementary Fig. 3b). In addition, iNSCs and GFP-iNSCs both expressed various HOX genes, including HOXA1-HOXC10 (Supplementary Fig. 3c). Moreover, both iNSCs and GFP-iNSCs differentiated into various subtypes of mature neurons, including GABA^+^ (GABAergic) and ISLET1^+^ (motor) neurons (Fig. 2c, Supplementary Fig. 3b).

### Survival and differentiation of grafted iNSCs in SCI models

Fibrin hydrogels encapsulating specific growth factors can improve the survival of transplanted NSCs, tissue repair, and functional recovery at the injured sites of SCI (Supplementary Fig. 4a)^5,6,26,27^. Cells should be smoothly injected over a defined period of time and gelled at a desired time point to minimize cell loss; therefore, we investigated the gelation time of various formulations. At lower concentrations of thrombin, the gelation time and pore size increased as the concentration of fibrinogen increased (Supplementary Fig. 4b, c). The viability of iNSCs in fibrin hydrogels was reduced at a high concentration of fibrinogen (30 mg/ml), which correlated with the results of the lactate dehydrogenase (LDH) assay (Supplementary Fig. 4d, e).

**Fig. 3:**
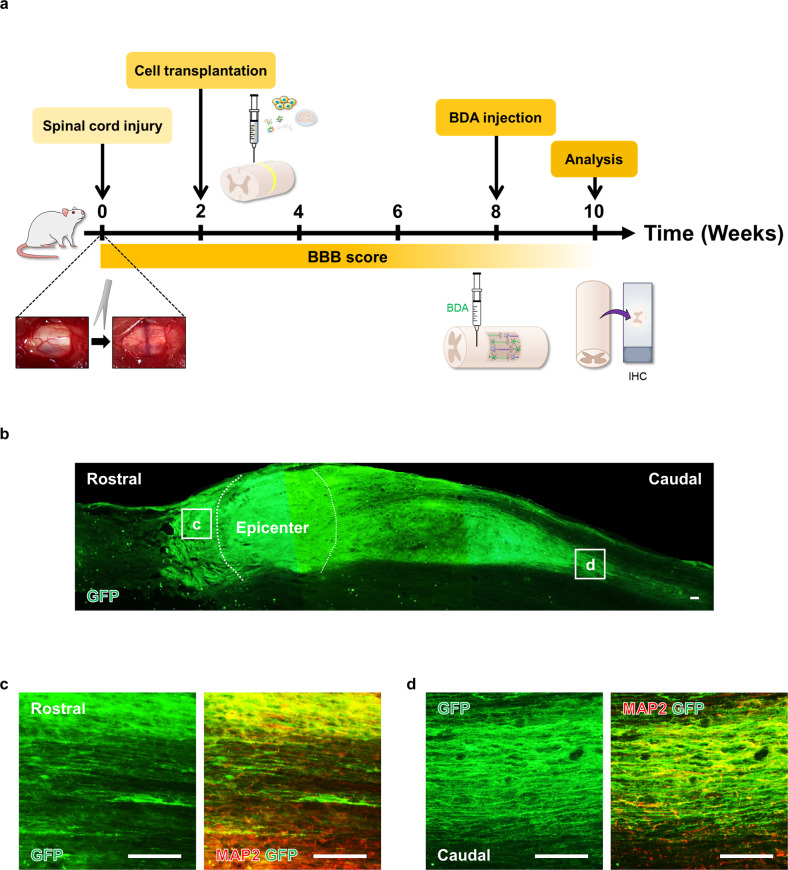
Survival of iNSCs at sites of spinal cord injury (SCI). **a** Schematic diagram of the experimental procedure. **b** Representative GFP fluorescent immunolabeled cells in horizontal sections from the GFP-iNSC Group 10 weeks after SCI. **c**, **d** High magnification of GFP-positive axons colabeled with MAP2 in the (**c**) rostral and (**d**) caudal regions to the T3 lesion site. Scale bars, 200 μm (**b**–**d**).

**Fig. 4:**
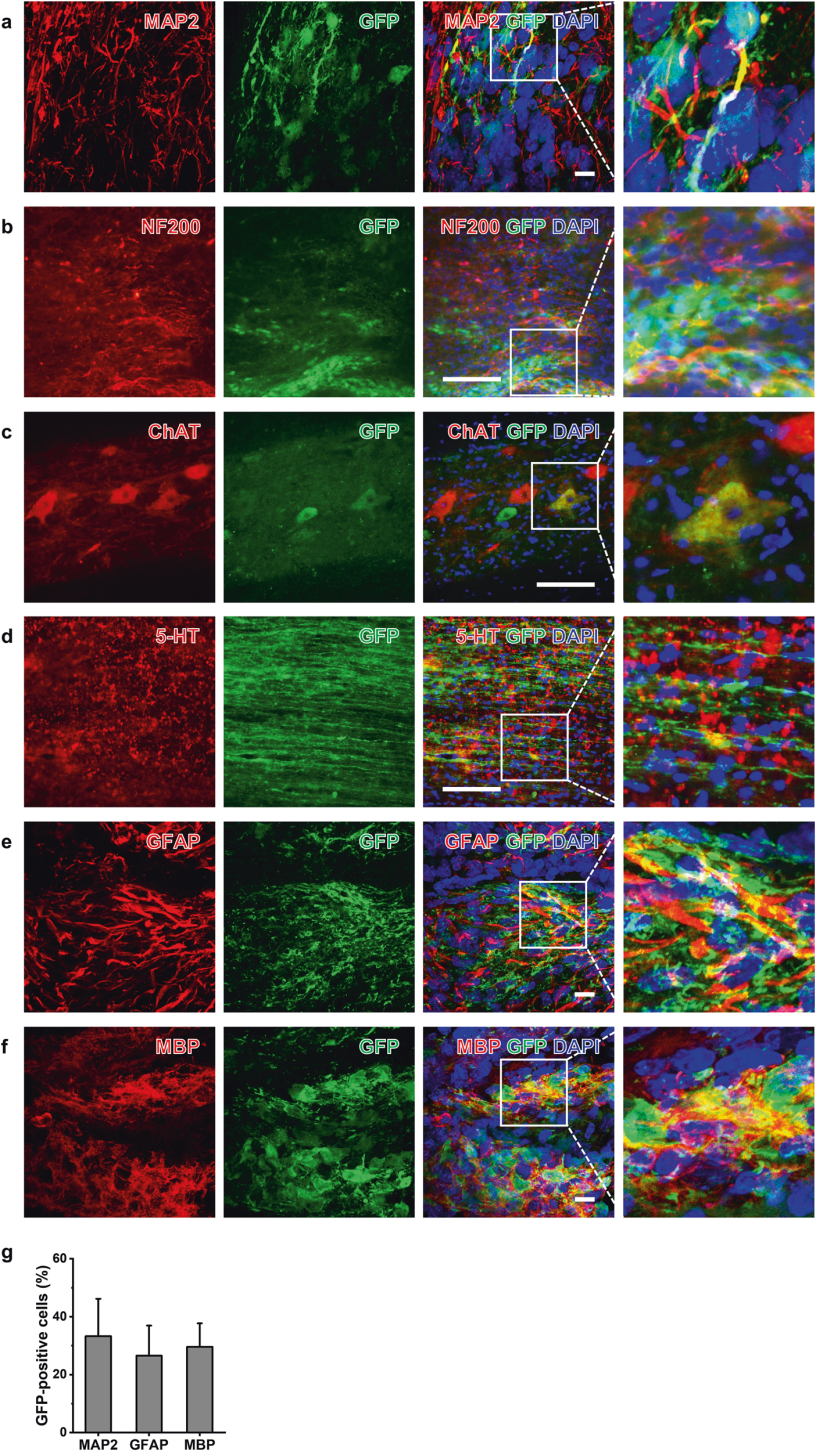
Differentiation of iNSCs in sites of SCI. **a**–**f** Images showing the expression of (**a**) MAP2, (**b**) neurofilament 200 (NF200), (**c**) choline acetyltransferase (ChAT), (**d**) 5-hydroxytryptamine (5-HT), (**e**) glial fibrillary acidic protein (GFAP), and (**f**) myelin basic protein (MBP) at the transplant site of the GFP-iNSC Group 10 weeks after SCI. Nuclei were counterstained with DAPI. **g** Quantification of the proportion of iNSC differentiation. Scale bars, 10 μm (**a**, **e**, and **f**); 200 μm (**b**–**d**).

To examine whether iNSCs generated neurons and glial cells in the injured spinal cord, we grafted GFP-iNSCs (Supplementary Fig. 3a) into the T3 spinal cord of Sprague–Dawley (SD) rats with severe crush injury (Fig. 3a)^36^. Two weeks after injury, rats were randomly separated into three groups: the SCI only group (*n* = 9); the iNSC group (*n* = 15), which included rats transplanted with iNSCs (*n* = 10) or GFP-iNSCs (*n* = 5); and the ESC-NSC group (*n* = 10). Cells were harvested and grafted into the lesion site. At 8 weeks posttransplantation, GFP^+^ cells survived and occupied most of the lesion cavity, with a large number of GFP^+^ cells extending into the host spinal cord in both the rostral and caudal directions over remarkably long distances (Fig. 3b), suggesting that human axons had branches that penetrated the host spinal cord. Graft-derived cells expressed a mature neuron marker (MAP2) (Fig. 3c, d), a neuronal fiber marker (NF200), a spinal motor neuron marker (choline acetyltransferase, ChAT), and a serotonergic neuronal marker (5-HT) (Fig. 4a–d). Grafts also expressed a mature astrocyte marker (GFAP) and a mature oligodendrocyte marker (MBP) at the same location as GFP-iNSCs (Fig. 4e, f), indicating that iNSCs differentiated into neurons, astrocytes, and oligodendrocytes in vivo (Fig. 4a–g). We obtained similar results in the ESC-NSC group (Supplementary Fig. 5). These results indicate that iNSCs survived for at least 2 months posttransplantation and retained their differentiation capacity in vivo.

### Graft-to-host connectivity

**Fig. 5:**
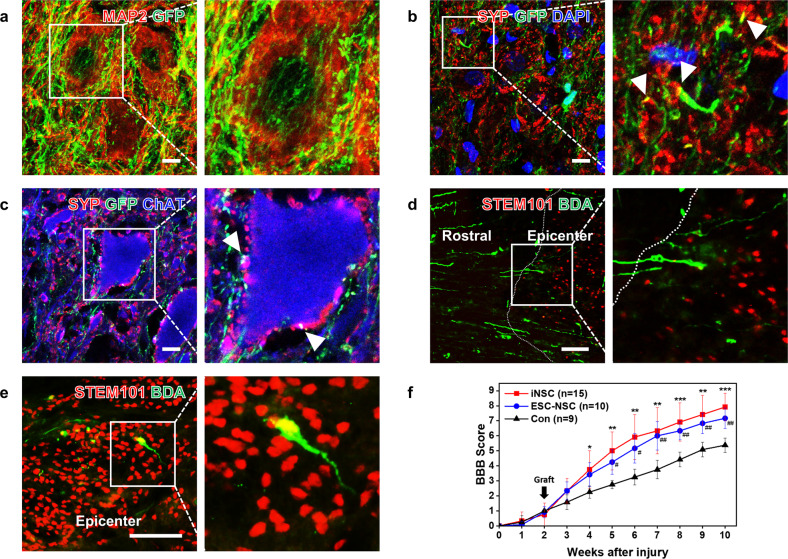
Graft-to-host connectivity and hindlimb locomotion. **a** GFP-labeled axons wrapped around MAP2^+^ neurons and dendrites. **b** Coexpression of GFP and synaptophysin (SYP) (arrowheads) in grafted cells. **c** GFP-labeled axons colocalized with SYP in contact with dendrites of ChAT^+^ host motor neurons (arrowheads). **d**, **e** Propriospinal axons labeled with biotinylated dextran amine (BDA) regenerated into iNSC grafts and human nuclei were stained with human-specific STEM101. **f** Comparison of the mean BBB score in the SCI only (Con), iNSC, and ESC-NSC groups. Data are represented as the mean ± SD. *Denotes a statistically significant difference between the Con and iNSC groups, *p < 0.05, **p < 0.01 *and* ***p < 0.001. ^#^Denotes a statistically significant difference between the Con and ESC-NSC groups, ^#^*p* < 0.05, ^##^*p* < 0.01 *and* ^###^*p* < 0.001. Scale bars, 10 μm (**a**–**c**); 200 μm (**d**, **e**).

An extraordinary number of human axons extended from grafted GFP-iNSCs into the host spinal cord over long distances (Fig. 3b). Furthermore, GFP-labeled axons grew out from the graft and colocalized with MAP2 (Fig. 3c, d). GFP-labeled human axons in the host spinal cord formed presynaptic bouton-like terminals that colocalized with neurons positive for the presynaptic marker synaptophysin (SYP) (Fig. 5a, b). Colocalization of GFP^+^ and SYP^+^ neurons with ChAT^+^ motor neurons was also observed (Fig. 5c). To investigate whether the host axons could stretch to the grafted iNSCs, we performed anterograde tracing of the propriospinal tract with biotinylated dextran amine (BDA) in SCI rats at 2 weeks before sacrifice^37^. BDA-labeled host axons penetrated iNSC grafts in spinal cord lesion sites and colocalized with STEM101, indicating that host-to-graft connectivity was achieved (Fig. 5d, e). Collectively, these findings demonstrate that grafted iNSC-derived axons projected from the site of SCI into the host spinal cord with synapse formation.

### Functional and behavioral outcomes

We evaluated motor behaviors weekly using the Basso, Beattie, and Bresnahan (BBB) open field locomotor scale^38^. Hindlimb locomotion was severely impaired after T3 crush SCI in SD rats and gradually recovered up to 10 weeks (Fig. 5f). At 10 weeks post-SCI (8 weeks after cell transplantation), the mean BBB score was significantly increased in the iNSC group and almost reached 8, indicating that plantar support of the paw without weight bearing was observed (Fig. 5f)^5^. In contrast, BBB scores were lower in the SCI only group, and these rats exhibited extensive movement of only one joint of the hindlimb, scored as 5 (Fig. 5f). These findings are consistent with the beneficial effects of NSC transplantation after SCI^5,6,26,27^.

### Locomotor recovery following transplantation of iNSCs in nude rats with SCI

**Fig. 6:**
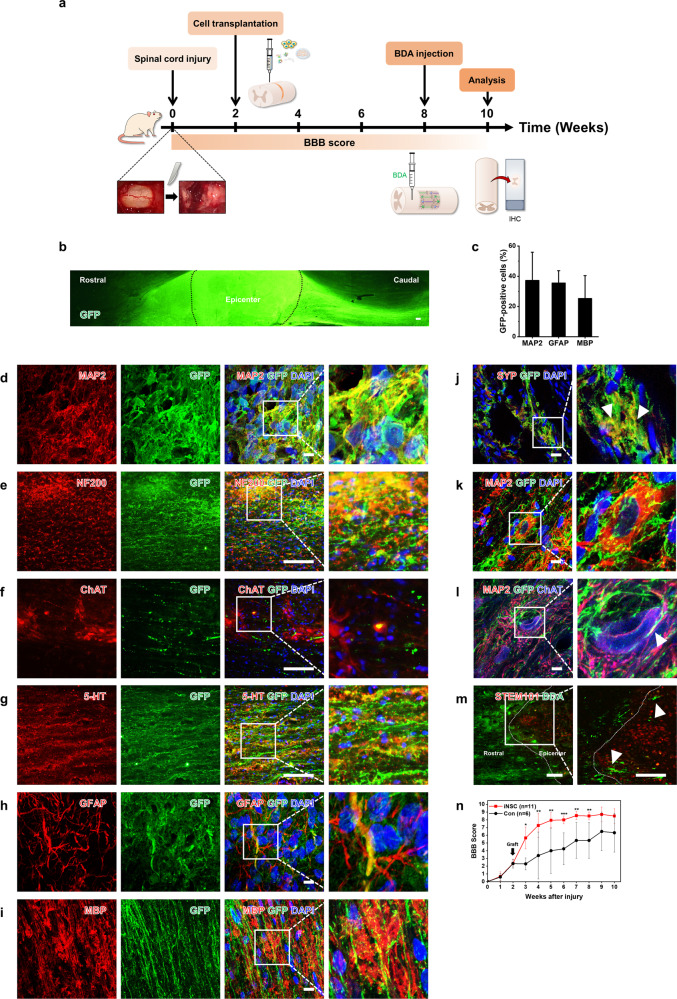
Improvement of locomotor recovery by iNSC grafts after SCI in nude rats. **a** Overview of the completely transected spinal cord of nude rats. **b** Representative GFP fluorescent immunolabeled cells in horizontal sections from the GFP-iNSC Group 10 weeks after SCI. **c** Quantification of the proportion of iNSC differentiation. **d**–**i** Images showing the expression of (**d**) MAP2, (e) NF200, (**f**) ChAT, (**g**) 5-HT, (**h**) GFAP, and (**i**) MBP at the transplant site of the GFP-iNSC Group 10 weeks after SCI. **j** Coexpression of GFP and SYP (arrowheads) in grafted cells. **k**, **l** Human axon terminals in contact with dendrites of host neurons (MAP2^+^ or ChAT^+^). **m** Propriospinal axons labeled with BDA regenerated into iNSC grafts. **n** Comparison of the mean BBB score in the vehicle (Con) and iNSC groups. Data are represented as the mean ± SD. *Denotes a statistically significant difference between the Con and iNSC groups, *p < 0.05, **p < 0.01 *and* ***p < 0.001. Scale bars, 200 μm (**b**, **e**–**g**, **m**); 10 μm (**d**, **h**–**l**).

Autoimmune T cells play destructive roles in a complex secondary injury cascade after SCI, as demonstrated by comparing the recovery of locomotor function between T-cell-deficient athymic nude rats (Crl:NIH-*Foxn1*^*rnu*^) and immunocompetent SD rats with SCI^39^. Importantly, cellular reprogramming is a powerful tool to produce immune-privileged patient-specific cells for autologous transplantation^19,40^. We evaluated the therapeutic effect of iNSCs on immunodeficient nude rats using a completely transected SCI model (Fig. 6a)^5,6^. Rats were randomly divided into two groups, the vehicle group (*n* = 6) and the iNSC group (*n* = 11), which included rats transplanted with iNSCs (*n* = 5) or GFP-iNSCs (*n* = 6). Cells were transplanted into the T3 lesion site at 2 weeks after the initial injury. Animals were perfused for anatomical analysis at 8 weeks post-transplantation. GFP^+^ cells survived, mostly filled the lesion site (Fig. 6b), and differentiated into neurons (MAP2^+^, NF200^+^, ChAT^+^, and 5-HT^+^), astrocytes (GFAP^+^), and oligodendrocytes (MBP^+^) (Fig. 6c–i). Similar to the findings made in SD rats with crush injury, a high density of GFP-labeled axons extended outward from the grafts toward the caudal and rostral host spinal cord over long distances (Fig. 6b). Bouton-like terminals formed in human axons coexpressing GFP and SYP (Fig. 6j) with close contact with dendrites of host neurons (MAP2^+^ or ChAT^+^) (Fig. 6k, l). Moreover, anterograde tracing analysis by BDA labeling showed that host propriospinal tract axons penetrated the grafts (Fig. 6m). BBB scoring revealed that hindlimb locomotor function was lost after complete transection injury in nude rats (Fig. 6n), and the mean BBB scores were significantly improved during the first 5 weeks after iNSC transplantation (2–7 weeks postinjury) compared with the vehicle group (Fig. 6n). Taken together, these results suggest that iNSCs can extensively differentiate into not only neurons but also astrocytes and oligodendrocytes with long-distance axonal growth, promote functional connectivity, and thus help to improve locomotor functions.

## Discussion

Autologous stem cell therapy has substantial advantages in terms of the potential risks of graft-versus-host disease, immune rejection, life-threatening complications, and opportunistic infections^41,42^. Recent studies using a nonhuman primate model of Parkinson’s disease demonstrated that autologous transplantation of iPSC-derived neural cells effectively improves motor recovery without immunosuppression and immunological rejection^43–45^. iPSCs were first established by Yamanaka and colleagues, and these personalized stem cells hold great promise in the fields of regenerative medicine, pathology research, and drug screening^46,47^. Although many studies have sought to develop safe and efficient methods for generating iPSCs in an integration-, virus-, and/or animal-free manner, several challenges, such as poor time and cost efficiency and the potential risk of tumorigenicity, remain to be overcome before these cells can be clinically applied on a large scale^47,48^.

Direct cellular reprogramming is an alternative approach to generate target cell types that bypass the intermediate pluripotent stage. It has advantages over other approaches for the generation of patient-specific cells and further therapeutic transplantation in terms of time and cost efficiency and tumorigenicity risk^19,40,47,48^. In addition to expandable and transplantable stem cells as precursors for nerve regeneration, cell survival and engraftment are critical for the successful translation of stem cell transplantation. Here, we demonstrated that diverse somatic cells can be directly reprogrammed into iNSCs that lack transgenes in their cytosol and genome and that these cells are similar to human ESC-NSCs in terms of their morphology, biological characteristics, in vitro/vivo differentiation capacity, and global gene expression^28^. Moreover, the reprogramming efficiency of iNSCs generated from UCs was similar to that of iNSCs generated from fibroblasts (0.025 ± 0.002%) (Supplementary Fig. 2a). More importantly, after transplantation of iNSCs into the brains of nude mice, tumor formation was not observed over 4 months^28^, suggesting that iNSCs are a transplantable cell source for SCI management. NSC transplantation has provided encouraging results in preclinical models and completed or ongoing clinical trials of SCI^5,6,8–10,21,27^.

**Fig. 7:**
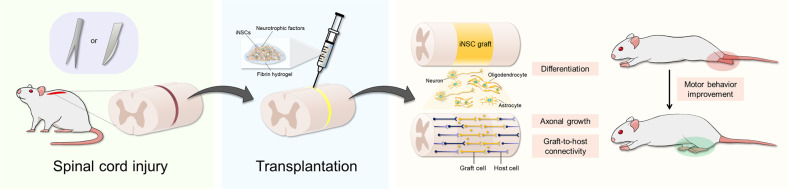
Schematic illustration. Schematic illustration of hydrogel-assisted transplantation employing transgene-free iNSCs in SCI rat models.

Neural regeneration with recruitment of stem cells can be accomplished by protecting against a series of secondary injuries, repopulating the tissue with neural lineage cells, regrowing large numbers of axons, rebuilding neural circuits, and thus functionally restoring the damaged spinal cord. Related studies have shown that when human NSCs are transplanted into the injured lesion of SCI, fibrin gels containing a growth factor cocktail allow these cells to survive for a long time and improve functional recovery^5,6,9,26^. In addition, large numbers of axons can extend from NSCs into the host spinal cord over long distances, which supports host-graft connectivity. In the present study, the therapeutic functions of iNSCs were assessed in SD rats with severe crush injury and nude rats with completely transected SCI. In agreement with previous studies^13,14,17,42^, iNSCs generated mature neuronal cell types and glial cell types and achieved extensive axonal growth beyond the lesion site for 8 weeks post-transplantation (Figs. 3, 4 and 6), leading to the formation of synaptic connections between host and graft neurons (Figs. 5, 6). Compared with the SCI only group, human iNSC grafts significantly improved BBB scores, which reflect all joint movements of the hindlimbs and plantar support without weight-bearing (Fig. 7).

Our iNSCs could be an alternative and optimal cell type for future clinical application to regenerate the spinal cord in patients with spinal cord diseases, including SCI. It has been reported that regional restriction of spinal cord NSCs can efficiently provide host spinal regeneration into the injury site^8,9,27^. In agreement with this, our iNSCs (both iNSCs and GFP-iNSCs) expressed spinal cord NSC markers (Supplementary Fig. 3c) and differentiated motor neuron markers (ISLET1 and ChAT) (Figs. 2c, 4c and 6f). Future studies will examine the therapeutic effects of iNSCs over a long-term period in SCI rat models and are warranted to investigate the impact of iNSCs on functional evaluations in large animal SCI models and other spinal cord diseases, including amyotrophic lateral sclerosis and spinal muscular atrophy.

In conclusion, proof-of-concept studies have demonstrated the therapeutic potential of transplanting personalized stem cells in various diseases and disorders that have long been considered incurable. However, the safety, differentiation, and functionality of these cells in vivo must be addressed before clinical trials are conducted. In the present study, we assessed the engraftment, lineage-specific differentiation, and axonal outgrowth of transgene-free iNSCs derived from human UCs and the connectivity between host and graft neurons in vivo. Our findings suggest that iNSCs are a safe and efficient tool for personalized autologous transplantation in SCI and various spinal cord diseases. Future studies are warranted to evaluate the impact of iNSC grafts on functional evaluations in nonhuman primate SCI models, and clinical evaluation will be pursued.

## Supplementary information

Supplementary information

## Data Availability

The data that support the findings of this study are available from the corresponding author upon reasonable request.

## References

[1] Pereira IM, Marote A, Salgado AJ, Silva NA (2019). Filling the gap: neural stem cells as a promising therapy for spinal cord injury. Pharmaceuticals (Basel).

[2] Shao A, Tu S, Lu J, Zhang J (2019). Crosstalk between stem cell and spinal cord injury: pathophysiology and treatment strategies. Stem Cell Res. Ther.

[3] Fischer I, Dulin JN, Lane MA (2020). Transplanting neural progenitor cells to restore connectivity after spinal cord injury. Nat. Rev. Neurosci..

[4] Takagi Y (2016). History of neural stem cell research and its clinical application. Neurol. Med. Chir. (Tokyo).

[5] Lu P (2012). Long-distance growth and connectivity of neural stem cells after severe spinal cord injury. Cell.

[6] Lu P (2014). Long-distance axonal growth from human induced pluripotent stem cells after spinal cord injury. Neuron.

[7] Sharp KG, Yee KM, Steward O (2014). A re-assessment of long distance growth and connectivity of neural stem cells after severe spinal cord injury. Exp. Neurol..

[8] Kadoya K (2016). Spinal cord reconstitution with homologous neural grafts enables robust corticospinal regeneration. Nat. Med..

[9] Rosenzweig ES (2018). Restorative effects of human neural stem cell grafts on the primate spinal cord. Nat. Med..

[10] Dulin JN (2018). Injured adult motor and sensory axons regenerate into appropriate organotypic domains of neural progenitor grafts. Nat. Commun..

[11] Hofstetter CP (2005). Allodynia limits the usefulness of intraspinal neural stem cell grafts; directed differentiation improves outcome. Nat. Neurosci..

[12] Cummings BJ (2005). Human neural stem cells differentiate and promote locomotor recovery in spinal cord-injured mice. Proc. Natl. Acad. Sci. USA.

[13] Plemel JR (2011). Platelet-derived growth factor-responsive neural precursors give rise to myelinating oligodendrocytes after transplantation into the spinal cords of contused rats and dysmyelinated mice. Glia.

[14] Uchida N (2000). Direct isolation of human central nervous system stem cells. Proc. Natl. Acad. Sci. USA.

[15] Jain M, Armstrong RJ, Tyers P, Barker RA, Rosser AE (2003). GABAergic immunoreactivity is predominant in neurons derived from expanded human neural precursor cells in vitro. Exp. Neurol..

[16] Casarosa S, Bozzi Y, Conti L (2014). Neural stem cells: ready for therapeutic applications?. Mol. Cell. Ther..

[17] Mothe, A. & Tator, C. H. Isolation of neural stem/progenitor cells from the periventricular region of the adult rat and human spinal cord. *J. Vis. Exp*. **1**, e52732 (2015). 10.3791/52732 PMC4542570 26067928

[18] Tang Y, Yu P, Cheng L (2017). Current progress in the derivation and therapeutic application of neural stem cells. Cell Death Dis..

[19] Xu J, Du Y, Deng H (2015). Direct lineage reprogramming: strategies, mechanisms, and applications. Cell Stem Cell.

[20] Wang H, Yang Y, Liu J, Qian L (2021). Direct cell reprogramming: approaches, mechanisms and progress. Nat. Rev. Mol. Cell Biol..

[21] Curtis E (2018). A first-in-human, Phase I study of neural stem cell transplantation for chronic spinal cord injury. Cell Stem Cell.

[22] Iwasaki M (2014). Synergistic effects of self-assembling peptide and neural stem/progenitor cells to promote tissue repair and forelimb functional recovery in cervical spinal cord injury. Biomaterials.

[23] Shetty AK, Hattiangady B (2016). Grafted subventricular zone neural stem cells display robust engraftment and similar differentiation properties and form new neurogenic niches in the young and aged hippocampus. Stem Cells Transl. Med..

[24] Jin MC, Medress ZA, Azad TD, Doulames VM, Veeravagu A (2019). Stem cell therapies for acute spinal cord injury in humans: a review. Neurosurg. Focus.

[25] Anwar MA, Al Shehabi TS, Eid AH (2016). Inflammogenesis of secondary spinal cord injury. Front. Cell. Neurosci..

[26] Robinson J, Lu P (2017). Optimization of trophic support for neural stem cell grafts in sites of spinal cord injury. Exp. Neurol..

[27] Kumamaru H (2018). Generation and post-injury integration of human spinal cord neural stem cells. Nat. Methods.

[28] Kang PJ (2019). mRNA-driven generation of transgene-free neural stem cells from human urine-derived cells. Cells.

[29] Li W (2011). Rapid induction and long-term self-renewal of primitive neural precursors from human embryonic stem cells by small molecule inhibitors. Proc. Natl. Acad. Sci. USA.

[30] Wang Z (2022). High-strength and injectable supramolecular hydrogel self-assembled by monomeric nucleoside for tooth-extraction wound healing. Adv. Mater..

[31] Zhou T (2011). Generation of induced pluripotent stem cells from urine. J. Am. Soc. Nephrol.

[32] Zhou T (2012). Generation of human induced pluripotent stem cells from urine samples. Nat. Protoc..

[33] Zafarullah M, Jasoliya M, Tassone F (2020). Urine-derived epithelial cell lines: a new tool to model Fragile X Syndrome (FXS). Cells.

[34] Onifer SM, White LA, Whittemore SR, Holets VR (1993). In vitro labeling strategies for identifying primary neural tissue and a neuronal cell line after transplantation in the CNS. Cell Transplant.

[35] Vroemen M, Aigner L, Winkler J, Weidner N (2003). Adult neural progenitor cell grafts survive after acute spinal cord injury and integrate along axonal pathways. Eur. J. Neurosci..

[36] Anderson MA (2018). Required growth facilitators propel axon regeneration across complete spinal cord injury. Nature.

[37] Jin Y, Fischer I, Tessler A, Houle JD (2002). Transplants of fibroblasts genetically modified to express BDNF promote axonal regeneration from supraspinal neurons following chronic spinal cord injury. Exp. Neurol.

[38] Basso DM, Beattie MS, Bresnahan JC (1996). Graded histological and locomotor outcomes after spinal cord contusion using the NYU weight-drop device versus transection. Exp. Neurol..

[39] Potas JR (2006). Augmented locomotor recovery after spinal cord injury in the athymic nude rat. J. Neurotrauma.

[40] Grath A, Dai G (2019). Direct cell reprogramming for tissue engineering and regenerative medicine. J. Biol. Eng..

[41] Miller CB (1996). Impact of age on outcome of patients with cancer undergoing autologous bone marrow transplant. J. Clin. Oncol..

[42] Kusnierz-Glaz CR (1997). Influence of age on the outcome of 500 autologous bone marrow transplant procedures for hematologic malignancies. J. Clin. Oncol..

[43] Morizane A (2013). Direct comparison of autologous and allogeneic transplantation of iPSC-derived neural cells in the brain of a non-human primate. Stem Cell Rep..

[44] Hallett PJ (2015). Successful function of autologous iPSC-derived dopamine neurons following transplantation in a non-human primate model of Parkinson’s disease. Cell Stem Cell.

[45] Tao Y (2021). Autologous transplant therapy alleviates motor and depressive behaviors in parkinsonian monkeys. Nat. Med..

[46] Takahashi K, Yamanaka S (2006). Induction of pluripotent stem cells from mouse embryonic and adult fibroblast cultures by defined factors. Cell.

[47] Liu G, David BT, Trawczynski M, Fessler RG (2020). Advances in pluripotent stem cells: history, mechanisms, technologies, and applications. Stem Cell Rev. Rep..

[48] Yamanaka S (2020). Pluripotent stem cell-based cell therapy-promise and challenges. Cell Stem Cell.

