# Supplementary material for: Human induced neural stem cells support functional recovery in spinal cord injury models
Source: Exp Mol Med. 2023 Jun 1;55(6):1182–92. doi: 10.1038/s12276-023-01003-2 (PMC10318049; doi:10.1038/s12276-023-01003-2)
Supplement: Supplementary file 1 — Supplementary information [file 12276_2023_1003_MOESM1_ESM.pdf]

Supplementary Information

Human induced neural stem cells support functional recovery in spinal cord injury models

Daryeon Son[1, 2, †], Jie Zheng[1, 2, †], In Yong Kim[1, 2, †], Phil Jun Kang[1], Kyoungmin Park[1], Lia Priscilla[1], Wonjun Hong[1], Byung Sun Yoon[4], Gyuman Park[5], Jeong-Eun Yoo[5], Gwonhwa Song[2, *], Jang-Bo Lee[3, *], Seungkwon You[1, 2, *]

Affiliations

[1] Laboratory of Cell Function Regulation, Department of Biotechnology, College of Life Sciences and Biotechnology, Korea University, Seoul, 02841, Republic of Korea

[2] Institute of Animal Molecular Biotechnology, College of Life Sciences and Biotechnology, Korea University, Seoul, 02841, Republic of Korea

[3] Department of Neurosurgery, College of Medicine, Korea University Anam Hospital, Seoul, 02841, Republic of Korea

[4] Institute of Regenerative Medicine, STEMLAB, Inc., Seoul, 02841, Republic of Korea

[5] Institute of Future Medicine, STEMLAB, Inc., Seoul, 02841, Republic of Korea

[†] These authors contributed equally to this work

[*]Correspondence

Seungkwon You, Ph.D. (E-mail: bioseung@korea.ac.kr) (Lead Contact)

Jang-Bo Lee, M.D., Ph.D. (E-mail: jangbo@korea.ac.kr)

Gwonhwa Song, Ph.D. (E-mail: ghsong@korea.ac.kr)

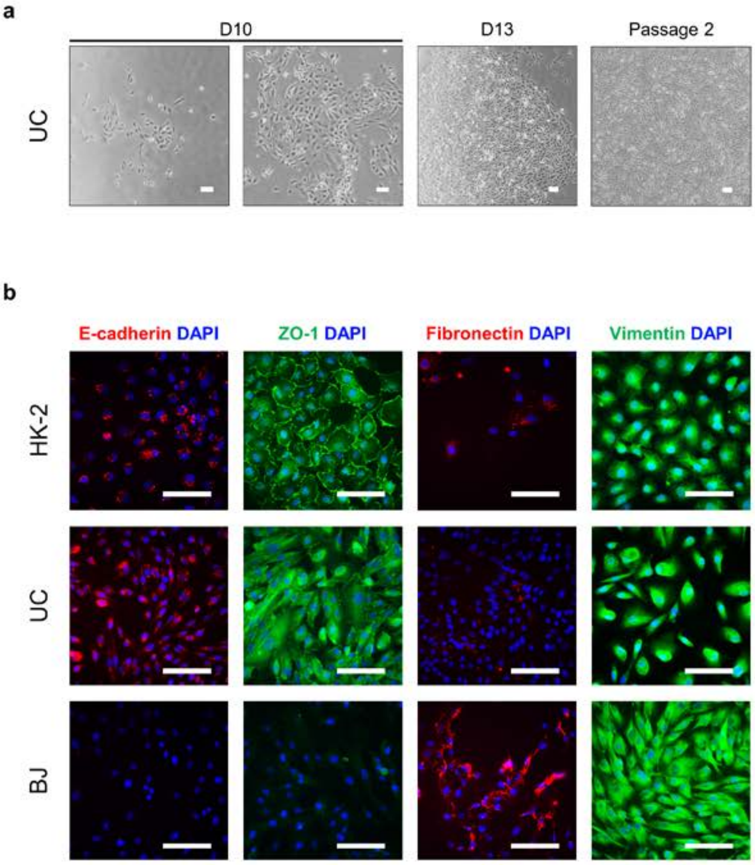
a
D10
D13
Passage 2
UC
b
E-cadherin DAPI
ZO-1 DAPI
Fibronectin DAPI
Vimentin DAPI
HK-2
UC
BJ

Supplementary Fig. 1 Characterization of urine cells (a) Representative images of urine cells (UCs) at day 10, 13 and passage 2. **(b)** Expression of epithelial markers (E-cadherin, ZO-1) and fibroblast markers (Fibronectin, Vimentin) in HK-2 cells, UCs, and BJ fibroblasts.

Scale bars, 200 μm (a-b)

a

iNSC line	Healthy donor 1	Healthy donor 2	Healthy donor 3	Healthy donor 4	Healthy donor 5	Patient 1	Patient 2	Patient 3
**NSC marker**	O	O	O	O	O	O	O	O
**Differentiation**	O	O	O	O	O	O	O	O
**Cell line authentication**	O	O	O	O	O	O	O	O
**Karyotype**	O	O	O	O	N.D.	O	O	O
**Mycoplasma contamination test**	O	O	O	O	O	O	O	O
**Reprogramming efficiency**	0.022% (n = 3)	0.030% (n = 3)	0.023% (n = 3)	Colony picking	Colony picking	Colony picking	Colony picking	0.025% (n =1)

b

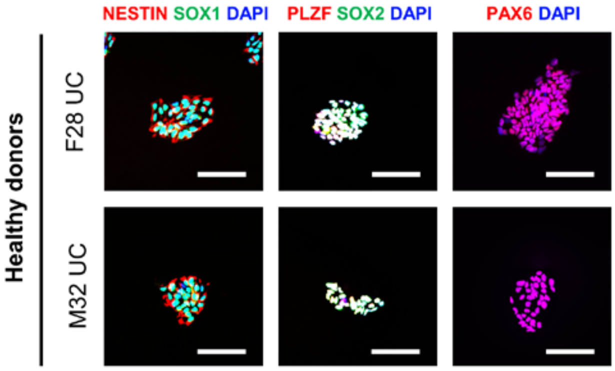

c

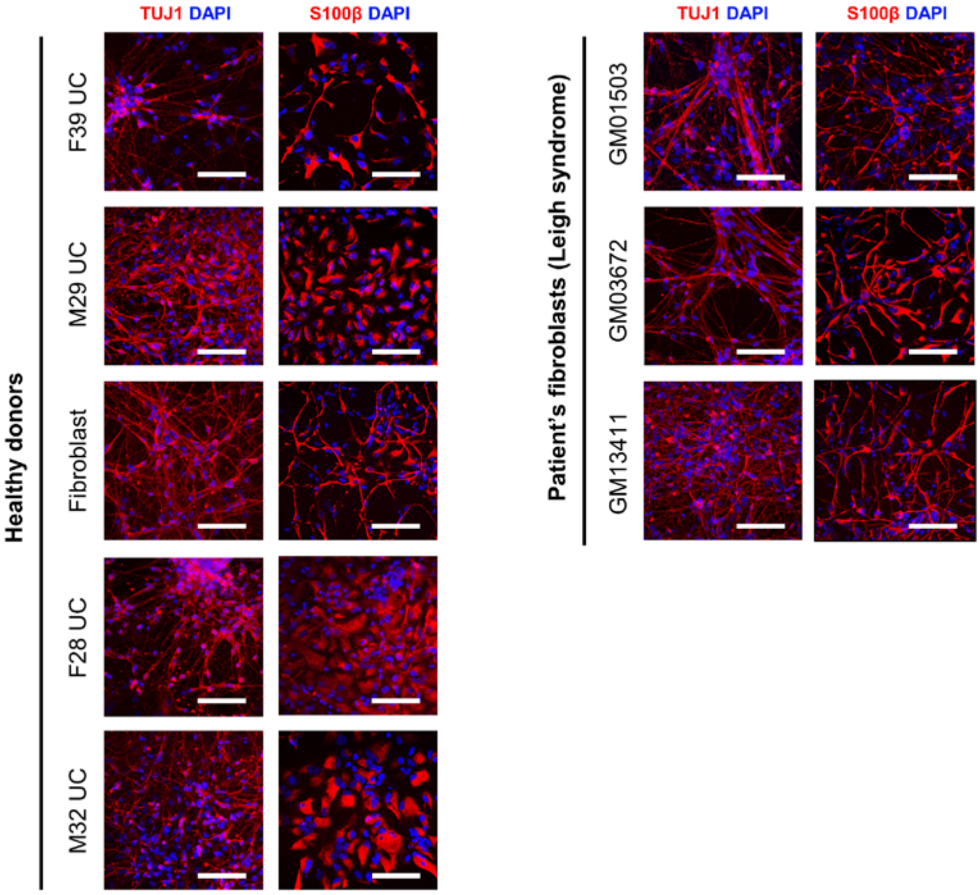

d

Healthy donor 1

Locus	Reference Database Profile		Sample Profile		Shared alleles #
	Database : F39 Uc		Sample Name : F39 iNSC		
D5S818	9	12	9	12	2
D13S317	8	13	8	13	2
D7S820	8	13	8	13	2
D16S539	10		10		1
vWA	17		17		1
TH01	6	7	6	7	2
TPOX	8		8		1
CSF1PO	10	12	10	12	2
AMEL	X		X		1
D3S1358	16	17	16	17	2
D21S11	29	30	29	30	2
D18S51	16		16		1
D8S1179	10	11	10	11	2
FGA	22.2	24	22.2	24	2
D2S1338	19	21	19	21	2
D19S433	12	14	12	14	2
Penta D	9		9		1
Penta E	14	20	14	20	2
Number of shared alleles					30
Total number of alleles in the reference database profile					30
% match					100.0%
**Result interpretation**					**Related**

Healthy donor 2

Locus	Reference Database Profile		Sample Profile		Shared alleles #
	Database : M29 Uc		Sample Name : M29 iNSC		
D5S818	9	14	9	14	2
D13S317	8		8		1
D7S820	8	12	8	12	2
D16S539	9	11	9	11	2
vWA	17	18	17	18	2
TH01	6	9	6	9	2
TPOX	11		11		1
CSF1PO	12		12		1
AMEL	X	Y	X	Y	2
D3S1358	15	17	15	17	2
D21S11	28.2	29	28.2	29	2
D18S51	12	17	12	17	2
D8S1179	13	14	13	14	2
FGA	21	28	21	28	2
D2S1338	17	24	17	24	2
D19S433	14	16	14	16	2
Penta D	9	11	9	11	2
Penta E	11	13	11	13	2
Number of shared alleles					33
Total number of alleles in the reference database profile					33
% match					100.0%
**Result interpretation**					**Related**

Healthy donor 3

Locus	Reference Database Profile		Sample Profile		Shared alleles #
	Database : BJ (Fibroblast)		Sample Name : BJ iNSC		
D5S818	12		12		1
D13S317	8	9	8	9	2
D7S820	11	12	11	12	2
D16S539	9	13	9	13	2
vWA	16	18	16	18	2
TH01	7	8	7	8	2
TPOX	10	11	10	11	2
CSF1PO	10	12	10	12	2
AMEL	X	Y	X	Y	2
D3S1358	14	16	14	16	2
D21S11	29		29		1
D18S51	17	19	17	19	2
D8S1179	9	11	9	11	2
FGA	22	23	22	23	2
D2S1338	16	20	16	20	2
D19S433	14		14		1
Penta D	12	13	12	13	2
Penta E	7	17	7	17	2
Number of shared alleles					33
Total number of alleles in the reference database profile					33
% match					100.0%
**Result interpretation**					**Related**

Healthy donor 4

Locus	Reference Database Profile		Sample Profile			Shared alleles #
	Database : F28 Uc		Sample Name : F28 iNSC			
D5S818	9	11	9	11		2
D13S317	8	9	8	9		2
D7S820	8	11	8	11		2
D16S539	10	11	10	11		2
vWA	14	17	14	17		2
TH01	6	9	6	9		2
TPOX	8	11	8	11		2
CSF1PO	10	12	10	12		2
AMEL	X		X			1
D3S1358	15	18	15	18		2
D21S11	29	33.2	29	33.2		2
D18S51	14	20	14	20		2
D8S1179	10	12	10	12		2
FGA	19	21	19	20	21	2
D2S1338	20	21	20	21		2
D19S433	13	14	13	14		2
Penta D	11	14	11	14		2
Penta E	8	11	8	11		2
Number of shared alleles						35
Total number of alleles in the reference database profile						35
% match						100.0%
**Result interpretation**						**Related**

Healthy donor 5

Locus	Reference Database Profile		Sample Profile		Shared alleles #
	Database : M32 Uc		Sample Name : M32 iNSC		
D5S818	9	11	9	11	2
D13S317	10	12	10	12	2
D7S820	8	11	8	11	2
D16S539	10	12	10	12	2
vWA	14	18	14	18	2
TH01	9		9		1
TPOX	8	11	8	11	2
CSF1PO	10	12	10	12	2
AMEL	X	Y	X	Y	2
D3S1358	15	16	15	16	2
D21S11	30		30		1
D18S51	13		13		1
D8S1179	13	15	13	15	2
FGA	23	25	23	25	2
D2S1338	19	20	19	20	2
D19S433	13	14.2	13	14.2	2
Penta D	9	12	9	12	2
Penta E	11	16	11	16	2
Number of shared alleles					33
Total number of alleles in the reference database profile					33
% match					100.0%
**Result interpretation**					**Related**

Patient 1

Locus	Reference Database Profile		Sample Profile		Shared alleles #
	Database : GM01503 Fibroblast		Sample Name : GM01503 iNSC		
D5S818	11	12	11	12	2
D13S317	8	11	8	11	2
D7S820	8	11	8	11	2
D16S539	12	13	12	13	2
vWA	18		18		1
TH01	7	9	7	9	2
TPOX	8	9	8	9	2
CSF1PO	11	13	11	13	2
AMEL	X		X		1
D3S1358	16	17	16	17	2
D21S11	28		28		1
D18S51	12	17	12	17	2
D8S1179	14	15	14	15	2
FGA	24	25	24	25	2
D2S1338	17	25	17	25	2
D19S433	13.2	14	13.2	14	2
Penta D	10	13	10	13	2
Penta E	12	15	12	15	2
Number of shared alleles					33
Total number of alleles in the reference database profile					33
% match					100.0%
**Result interpretation**					**Related**

Patient 2

Locus	Reference Database Profile		Sample Profile		Shared alleles #
	Database : GM03672 Fibroblast		Sample Name : GM03672 iNSC		
D5S818	11		11		1
D13S317	13	14	13	14	2
D7S820	9	10	9	10	2
D16S539	12	14	12	14	2
vWA	16	20	16	20	2
TH01	8	9.3	8	9.3	2
TPOX	8	11	8	11	2
CSF1PO	9	10	9	10	2
AMEL	X		X		1
D3S1358	14	16	14	16	2
D21S11	28	30	28	30	2
D18S51	13	16	13	16	2
D8S1179	8	13	8	13	2
FGA	23	24	23	24	2
D2S1338	16	18	16	18	2
D19S433	13	14	13	14	2
Penta D	13		13		1
Penta E	12		12		1
Number of shared alleles					32
Total number of alleles in the reference database profile					32
% match					100.0%
**Result interpretation**					**Related**

Patient 3

Locus	Reference Database Profile		Sample Profile		Shared alleles #
	Database : GM13411 Fibroblast		Sample Name : GM13411 iNSC		
D5S818	10	12	10	12	2
D13S317	10	11	10	11	2
D7S820	10.1	11	10.1	11	2
D16S539	9	12	9	12	2
vWA	17		17		1
TH01	9		9		1
TPOX	11		11		1
CSF1PO	10		10		1
AMEL	X	Y	X	Y	2
D3S1358	15	16	15	16	2
D21S11	30		30		1
D18S51	12	15	12	15	2
D8S1179	11	13	11	13	2
FGA	22	26	22	26	2
D2S1338	23	24	23	24	2
D19S433	14.2	15.2	14.2	15.2	2
Penta D	11	12	11	12	2
Penta E	13	16	13	16	2
Number of shared alleles					31
Total number of alleles in the reference database profile					31
% match					100.0%
**Result interpretation**					**Related**

e

Sample	MycoAlert ™ Ratio
Positive Sample	192.55
Negative Sample	0.86
Healthy donor 1	0.91

Sample	MycoAlert ™ Ratio
Positive Sample	192.55
Negative Sample	0.86
Healthy donor 2	0.78

Sample	MycoAlert ™ Ratio
Positive Sample	240.52
Negative Sample	0.82
Healthy donor 3	0.49

Sample	MycoAlert ™ Ratio
Positive Sample	210.5
Negative Sample	0.97
Healthy donor 4	0.68

Sample	MycoAlert ™ Ratio
Positive Sample	189.92
Negative Sample	0.83
Healthy donor 5	0.80

Sample	MycoAlert ™ Ratio
Positive Sample	240.52
Negative Sample	0.82
Patient 1	0.81

Sample	MycoAlert ™ Ratio
Positive Sample	240.52
Negative Sample	0.82
Patient 2	0.84

Sample	MycoAlert ™ Ratio
Positive Sample	240.52
Negative Sample	0.82
Patient 3	0.63

Ratio	Interpretation
< 1	Negative for mycoplasma
> 1.2	Mycoplasma contamination

Supplementary Fig. 2 Characterization of iNSCs (a) List of iNSCs. **(b)** Expression of NSC markers (NESTIN, SOX1, PLZF, SOX2, and PAX6) of iNSCs. **(c)** Expression of TUJ1 and S100β in differentiated iNSCs. **(d)** STR analysis of iNSCs. **(e)** Mycoplasma contamination test.

Scale bars, 200 μm

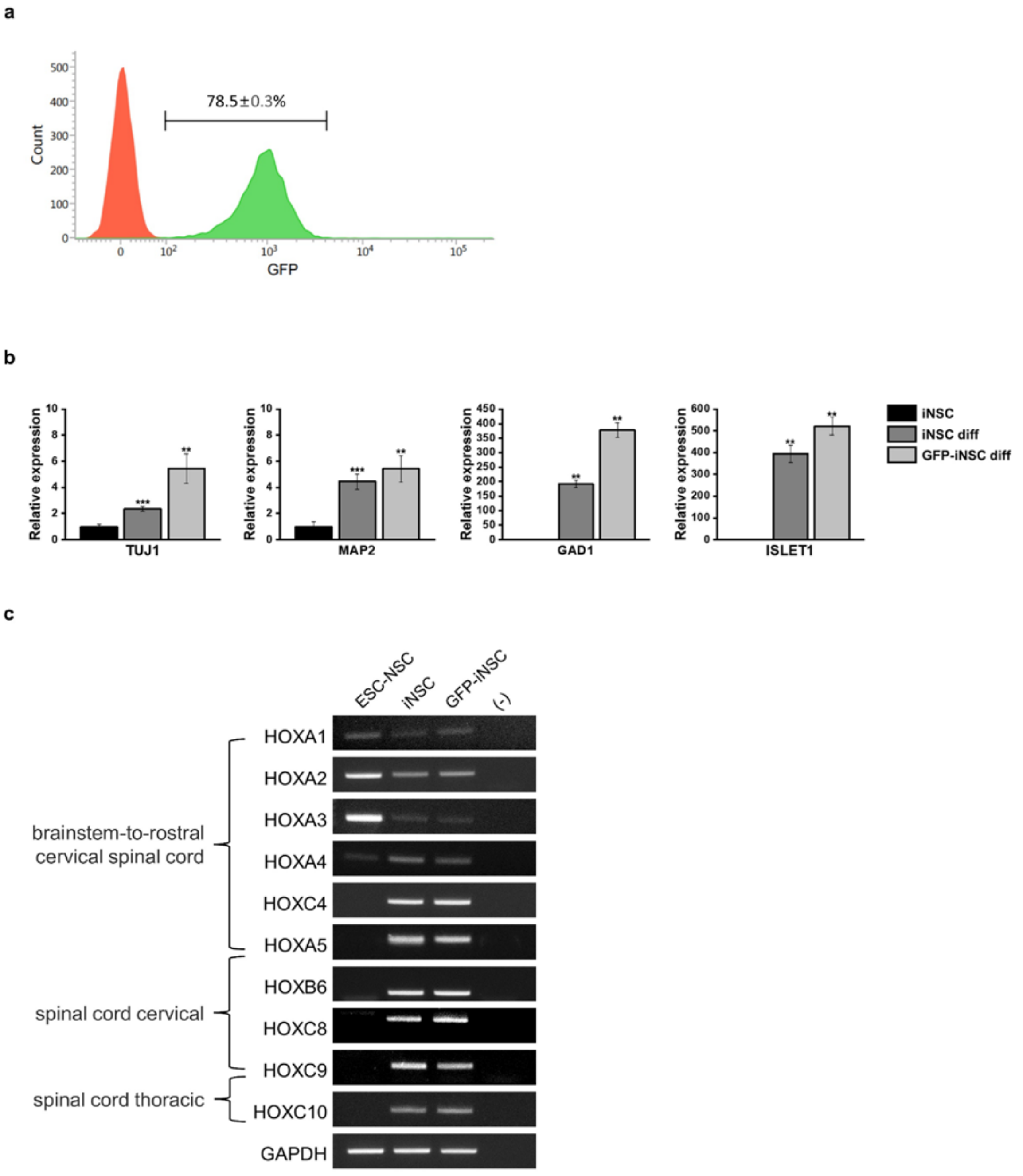

a
78.5±0.3%
Count
GFP
b
Relative expression
TUJ1
MAP2
GAD1
ISLET1
iNSC
iNSC diff
GFP-iNSC diff
c
ESC-NSC
iNSC
GFP-iNSC
(-)
HOXA1
HOXA2
HOXA3
HOXA4
HOXC4
HOXA5
HOXB6
HOXC8
HOXC9
HOXC10
GAPDH
brainstem-to-rostral cervical spinal cord
spinal cord cervical
spinal cord thoracic

Supplementary Fig. 3 Characterization of iNSCs and GFP-iNSCs (a) FACS analysis of GFP expression in GFP-iNSCs. **(b)** Real-time PCR analysis of neuronal markers (TUJ1, MAP2, GAD1 and ISLET1) in differentiated iNSCs and GFP-iNSCs. **(c)** RT-PCR for various HOX genes expression in iNSCs and GFP-iNSCs. Data are represented as mean ± SD. * denotes a statistically significant difference between the iNSC and iNSC-diff group, **p <0.05, **p <0.01 and ***p <0.001*.

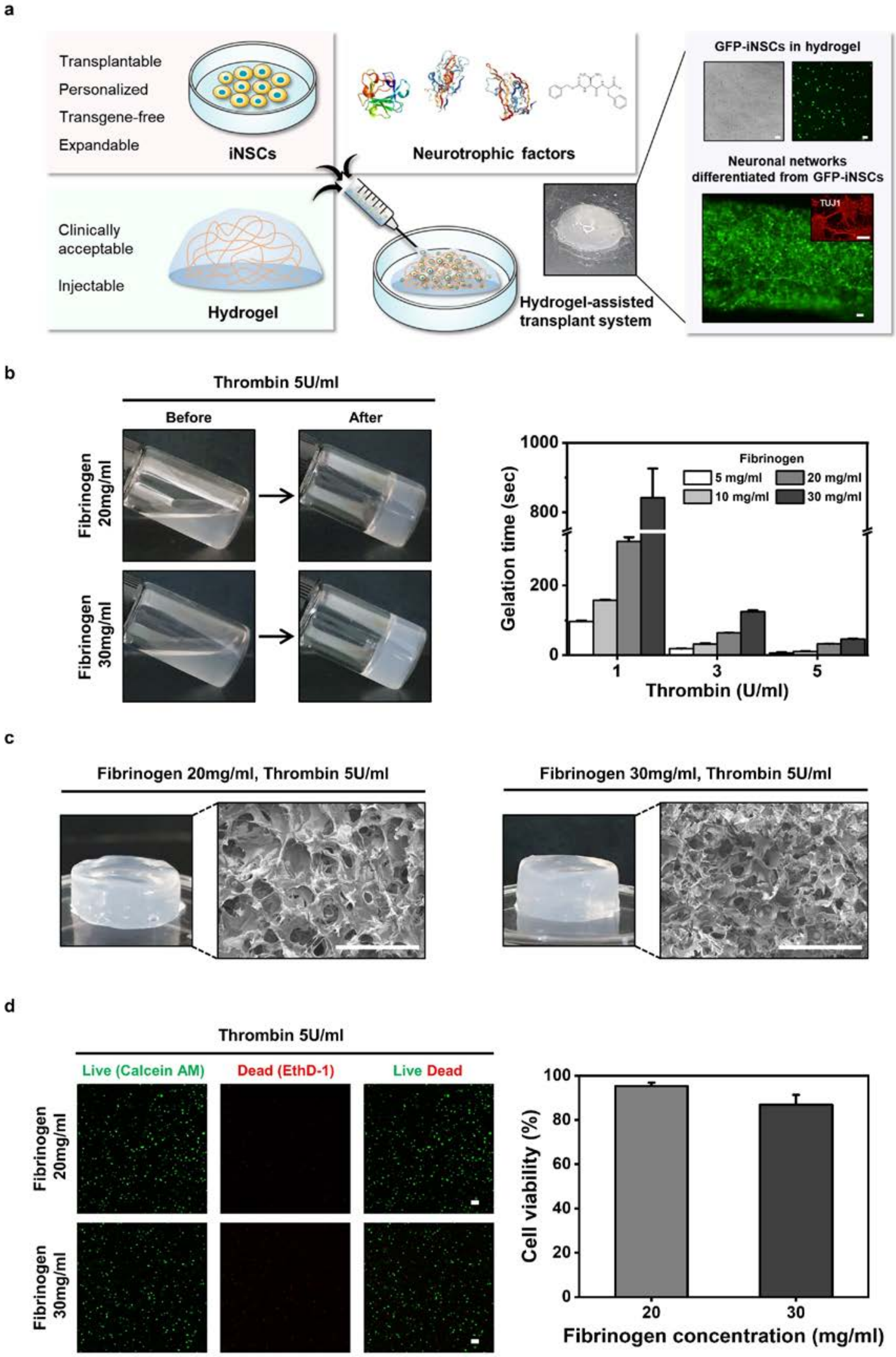
a
Transplantable
Personalized
Transgene-free
Expandable
iNSCs
Neurotrophic factors
GFP-iNSCs in hydrogel
Neuronal networks differentiated from GFP-iNSCs
TUJ1
Clinically acceptable
Injectable
Hydrogel
Hydrogel-assisted transplant system
b
Thrombin 5U/ml
Before
After
Fibrinogen 20mg/ml
Fibrinogen 30mg/ml
Fibrinogen
5 mg/ml
10 mg/ml
20 mg/ml
30 mg/ml
Gelation time (sec)
Thrombin (U/ml)
c
Fibrinogen 20mg/ml, Thrombin 5U/ml
Fibrinogen 30mg/ml, Thrombin 5U/ml
d
Thrombin 5U/ml
Live (Calcein AM)
Dead (EthD-1)
Live Dead
Fibrinogen 20mg/ml
Fibrinogen 30mg/ml
Cell viability (%)
Fibrinogen concentration (mg/ml)

e

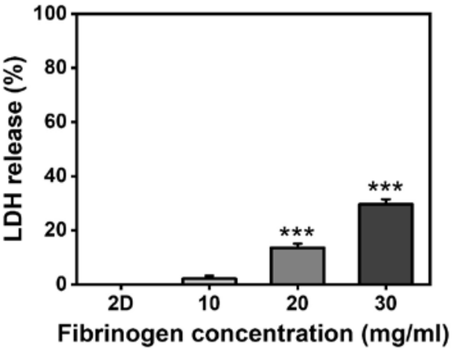

LDH release (%)
100
80
60
40
20
0
***
***
2D
10
20
30
Fibrinogen concentration (mg/ml)

Supplementary Fig. 4 Characterization and cytotoxicity of fibrin hydrogels (a) Schematic diagram of injectable fibrin hydrogel system. **(b)** Gelation time of fibrin hydrogels at different concentrations. **(c)** SEM images of fibrin hydrogels**. (d-e)** Representative images of live/dead staining and percentage of LDH release. Data are represented as mean ± SD. * denotes a statistically significant difference between the 2D and experiment group, *$*p < 0.05$, $**p < 0.01$ and $***p < 0.001$*.

Scale bars, 500 μm (c), 200 μm (d)

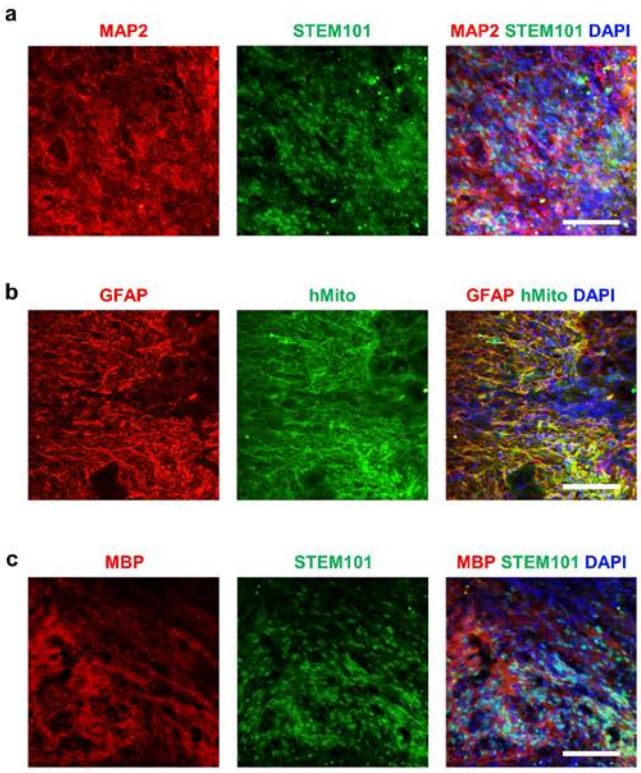
a
MAP2
STEM101
MAP2 STEM101 DAPI
b
GFAP
hMito
GFAP hMito DAPI
c
MBP
STEM101
MBP STEM101 DAPI

Supplementary Fig. 5 Survival and differentiation of ESC-NSCs in SD rats Images showed expression of **(a)** MAP2, **(b)** GFAP, and **(c)** MBP at the transplant site of ESC-NSCs group 10 weeks after SCI.

Scale bars, 200 μm (a-c)

Supplementary Table 1. Primers used in this study

Gene	Primer	Sequence (5'-3')
TUJ1	Forward	CTCAGGGGCCTTTGGACATC
	Reverse	CAGGCAGTCGCAGTTTTCAC
MAP2	Forward	CAACGGAGAGCTGACCTCA
	Reverse	CTACAGCCTCAGCAGTGACTA
GAD1	Forward	CAAACATTTATCAACATGCGCTTC
	Reverse	CTATGACACTGGAGACAAGGC
ISLET1	Forward	CGTGCCCGCTCCAAGGTGTATCA
	Reverse	CATTGGGCTGCTGCTGCTGGAGTT
HOXA1	Forward	TCCTGGAATACCCCATACTTAGCA
	Reverse	GCCGCCGCAACTGTTG
HOXA2	Forward	ACTCCTTTGACCAGGTGGTTTTGC
	Reverse	ACTTTCTTGCAGGCCTCATACTGC
HOXA3	Forward	ATTGCTCCAAAAATCTGCACGCGG
	Reverse	ATTCAGCAGGAAGCTAATGCTGGG
HOXA4	Forward	TCCCCATCTGGACCATAATAGG
	Reverse	GCAACCAGCACAGACTCTTAACC
HOXC4	Forward	GGGTGAATTTCAGGGGAAATGAGG
	Reverse	CTCAAACTGAACAGCTCTGAGAGG
HOXA5	Forward	TCTCGTTGCCCTAATTCATCTTTT
	Reverse	CATTCAGGACAAAGAGATGAACAGA
HOXB6	Forward	AGCAGAGCAAAATGCTCTTGTCCC
	Reverse	GAGGCTCCTCTTCTTACTTCTAGG
HOXC8	Forward	AGGAACCTGATGGAAACCTGAAGG
	Reverse	ATCAAACAGCGAAGGAGAGGAAGG
HOXC9	Forward	TAGAGTTAGTTCTACCCAGCGAGG
	Reverse	ACCTGGACCAAATACGATACAGGG
HOXC10	Forward	CTCACACACAGCATTCTGTTCTCC
	Reverse	ACACGAACACTAGCCGAACTTTCC
GAPDH	Forward	GTGGTCTCCTCTGACTTCAACA
	Reverse	CTCTTCCTCTTGTGCTCTTGCT

Supplementary Table 2. Primary antibodies used in this study

Antibody	Company	Catalog number	Host	Dilution
NESTIN	MilliporeSigma	MAB5326	Mouse	1:500
PLZF	R&D Systems	MAB2944	Mouse	1:200
SOX1	R&D Systems	AF3369	Goat	1:200
SOX2	R&D Systems	AF2018	Goat	1:200
PAX6	DSHB	PAX6	Mouse	1:200
TUJ1	BioLegend	801202	Mouse	1:200
MAP2	MilliporeSigma	AB5622	Rabbit	1:200
GABA	MilliporeSigma	A2052	Rabbit	1:200
ISLET1	DSHB	40.2D6	Mouse	1:200
S100β	MilliporeSigma	S2532	Mouse	1:200
GFP	Abcam	ab1218	Mouse	1:200
GFP	Abcam	ab260	Rabbit	1:200
GFAP	Invitrogen	13-0300	Rat	1:200
MBP	MilliporeSigma	MAB386	Rat	1:200
NF200	MilliporeSigma	N4142	Rabbit	1:200
ChAT	MilliporeSigma	AB144P	Goat	1:200
5-HT	ImmunoStar	24330	Rabbit	1:100
STEM101	Takara	Y40400	Mouse	1:100
Synaptophysin	Santa Cruz Biotechnology	SC17750	Mouse	1:200
Human Mitochondria	MilliporeSigma	MAB1273	Mouse	1:100
E-cadherin	BD Biosciences	610181	Mouse	1:100
ZO-1	Invitrogen	61-7300	Rabbit	1:100
Fibronectin	Abcam	ab6328	Mouse	1:100
Vimentin	MilliporeSigma	AB1620	Goat	1:200
